# Near-Resonant Imaging of Trapped Cold Atomic Samples

**DOI:** 10.6028/jres.101.057

**Published:** 1996

**Authors:** L. You, Maciej Lewenstein

**Affiliations:** Institute for Theoretical Atomic and Molecular Physics, Harvard-Smithsonian Center for Astrophysics, 60 Garden Street, MS 14, Cambridge, MA 02138; Commissariat à l’Energie Atomique, Centre d’Etudes deSaclay, Service de Photons, Atomes, et Molécules, Bâtiment 524, Gif-sur-Yvette 91191 Cedex, France

**Keywords:** Bose-Einstein condensation, light scattering, optical imaging, quantum statistics

## Abstract

We study the formation of diffraction patterns in the near-resonant imaging of trapped cold atomic samples. We show that the spatial imaging can provide detailed information on the trapped atomic clouds.

## 1. Introduction

With the recent observation of Bose-Einstein condensate (BEC) in both systems of trapped rubidium [1] and sodium [2] atoms, and the reported evidence of BEC in a system of lithium atoms [3], it has became urgent to study the diffraction patterns in the near-resonant imaging of cold atomic samples. This problem is especially important since it concerns the only straight forward *in situ* detecting scheme for the tiny atomic clouds undergoing evaporation cooling [4]. Optical imaging provides thus a possible diagnostics for the formation of BEC.

In the experiment at JILA by Anderson et al. [1], a *weakly interacting* BEC (according to the criteria set by the BEC research in homogeneous systems [5]) has been observed. Even though such a condensate is closer to an ideal noninteracting BEC than any other previous system which exhibits bosonic degeneracy, its kinetic properties differ significantly from the ideal gas case. It is now well established that for the particular state of ^87^Rb used at JILA the scattering length is positive [6] (i.e., atom-atom interactions are predominantly repulsive), while that of ^7^Li used by Bradley et al. is negative [7] (i.e., atom-atom interactions are attractive). The inhomogeneity introduced by a trap potential allows one to optimize the compression in phase space [8], but it strongly affects the boundary conditions for the system. This is one of the reasons why BEC in a trap is possible for atoms with a negative scattering length [9], whereas it is forbidden in a homogeneous system [10]. Indeed, in a trap the relation between stability of the BEC and the two-body interaction scattering length becomes more complex even at zero temperature [9,11,12].

To probe the presence of BEC and to study its unusual properties, we have to consider its interactions with the external world. Scattering of photons from a near resonant laser provides one of the most natural choices for such a measurement scheme [13].

The aim of this paper is to examine the problem of imaging BEC within a cold atomic sample by coherent scattering of a weak, near-resonant, continuous-wave laser field. Two separate studies are required for the full description of near-resonant imaging. First, a reliable theory of the weak field scattering has to be developed, and the near field pattern has to be calculated. Second, realistic calculation of the image formation process is needed. Such a calculation must necessarily take into account the presence of apertures, lenses etc. In this paper we combine the recently developed theory of coherent light scattering [13], with the theory of optical imaging.

The paper is arranged as follows: in Sec. 2 we briefly review the theories of weak light scattering off a BEC. We concentrate on the two approaches developed in Ref. [13]: the *on-shell* approximation, and Glauber’s *generalized diffraction theory*. Using these methods, we construct the near field distribution of the scattered light, and express the scattering off the condensate in terms of an optical transmission process with a complex transmission function including both phase shift (refraction) and amplitude attenuation (absorption). We compare the numerical results for this transmission function obtained using the two above mentioned approaches. In Sec. 3 we describe a simple imaging system consisting of a single ideal converging lens and an aperture in front of it. The impulse response function is derived in the paraxial approximation limit, and the expression for the field distribution in the image plane is given. This is the main result of this work. In Sec. 4 we analyse numerically the properties of the image using the parameters that correspond closely to those used in the JILA and Rice experiments [1, 3]. Finally, we conclude in Sec. 5.

## 2. Coherent Light Scattering

The literature on coherent light scattering from cold atomic clouds is already quite extensive (see Ref. [13], and references therein). Qualitatively, various effects are predicted, depending on the size and density of the atomic cloud, in particular, of the condensate. Large and dense condensates should lead to formation of *polaritons*, a gap in the excitation spectrum, and deflection, or even back reflection of the laser light from the atomic sample [14, 15]. Small condensates, on the other hand, should lead to a collective response of atoms, and a collectively broadened Lorentzian spectrum [16]. For not too large, and not too dense condensates (i.e., for situations corresponding to experimental conditions of Refs. [1, 3]), the theory developed by us in Refs. [13, 17], seems to be the most suitable. This theory does not lead to the formation of a gap in the spectrum, since the photon energy is larger than the appropriately defined optical potential. Photons are thus scattered primarily in the forward direction, but nevertheless are affected strongly by the medium. The scattering and absorption cross sections have nonLorentzian shapes [17], with an overall width determined by collective excitations (as in Ref. [16]),

$$\gamma_{\mathrm{eff}}≃3N\gamma /2{\left(k_{L}a\right)}^{2},$$ (1)

where *N* is the number of atoms, *γ* is a single atom spontaneous emission width, *k*_L_ = 2*π*/*λ* is the laser wavevector, *λ* is the laser wavelength, and *a* is the condensate size. The spectrum gradually narrows towards the resonance, and exhibits a cusp at the exact resonance with a width controlled by single atom dephasing processes and losses, such as spontaneous emission to uncondensed states, quantum diffusion etc.

Using the expressions derived in Ref. [13], we can construct the near field distribution. In the *on-shell* approximation [18], our starting point is a scattering equation valid in the weak field limit. For spherically symmetric density distributions, the asymptotic solution for the averaged photon operator *a_k__μ_* has the form

$$a_{k\mu }\left(t\to \infty \right)\to \left[\delta \left(k-k_{L}\right)\delta_{\mu \mu_{L}}+B\left(k,\mu \right)\delta \left(ck-\omega_{L}\right)\right]e^{-i\omega_{L}t},$$ (2)

where the first term describes the incident plane wave, while the second describes the process of elastic scattering with the scattering amplitude

$$B\left(k,\mu \right)=\sum_{l=0}^{\infty }\frac{c}{k_{L}^{2}}B_{l}\left(k_{L}\right)P_{l}\left(\cos\theta \right)\left(2l+1\right)/4\pi ,$$ (3)

where *P_l_* (*x*) are the Legendre polynomials. We use here the coordinate system in which ***k*** = *k_L_* (sin*θ* cos*ϕ*, sin*θ* sin*ϕ*, cos*θ*), ***k****_L_* = *k_L_* (0, 0, 1), and the polarization vector *ε_μL_* = (1, 0, 0), so that ***z*** defines the optical axis. The coefficients *B_l_* (*k_L_*) can be well approximated by the expression

$$B_{l}\left(k_{L}\right)\approx -\frac{\gamma_{L}^{l}}{\left[\Gamma +\gamma_{L}^{l}-i\left(\omega_{L}-\omega_{0}\right)\right]},$$ (4)

where $\gamma_{L}^{l}=12\pi \gamma \int_{0}^{\infty }r^{2}drn(r)j_{1}^{2}(k_{L}r)$, with *n*(*r*) being the density distribution of cold atoms in a trap and *j_l_* being the spherical Bessel function of order *l*. *Γ* is an effective single atom dephasing rate which accounts for spontaneous emission to the uncondensed states, quantum diffusion of the excited wave packet, etc. Evidently, in typical situation *Γ* is larger than both the quantum diffusion rate and *γ* [13]. *ω*_L_ and *ω*_0_ are respectively the probe laser frequency and the atomic resonance frequency.

The reconstruction of the field distribution at any spatial point can be made according to

$$\begin{array}{c} E_{\mathrm{OS}}\left(r\right)\propto -\sum_{\mu }\int dk♇\left(k\right)\;\varepsilon_{\mu }a_{k\mu }e^{ik\cdot r}\\ =\varepsilon_{0}\left(\rho \right)e^{ik_{L}z}\psi_{\mathrm{OS}}\left(\rho ,z\right), \end{array}$$ (5)

In the above expression *ε*_0_(***ρ***) is the probe field amplitude, whereas ♇(k) is the dipole coupling constant, which is a slowly varying function of *k* and is related to *γ* by $\gamma =\left(8\pi^{2}k_{L}^{2}/3c\right){\left|♇\left(k_{L}\right)2\right|}^{2}$. We assume that the polarization of the field *E*_OS_(***r***) is approximately equal to the polarization of the probe field, so vector notation can be suppressed.

The scaled near field amplitude is given by

$$\begin{array}{c} \psi_{\mathrm{OS}}\left(\rho ,z\right)=1+\sum_{l=0}^{\infty }\frac{2l+1}{2}\;B_{l}\;(k_{L})\\ \times \int_{0}^{\pi }\sin\;\theta \;d\;\theta e^{ik_{L}z\left(\cos\theta -1\right)}\;J_{0}\left(k_{L}\rho \sin\theta \right)\;P_{l}\left(\;\cos\theta \right), \end{array}$$ (6)

where *J*_0_ is the Bessel function of order 0. In the *generalized diffraction theory* (GDT) [19], the solution obtained after the propagation through the cold atom system can be expressed in a form analogous to Eq. (5).

$$E_{\mathrm{GDT}}\left(r\right)=\varepsilon_{0}\left(\rho \right)e^{ik_{L}z}\psi_{\mathrm{GDT}}\left(\rho ,z\right),$$ (7)

with

$$\psi_{\mathrm{GDT}}\left(\rho ,z\right)=e^{-i\int_{-\infty }^{z}V\left(\rho ,z\prime \right)dz\prime },$$ (8)

and the optical potential defined as

$$V\left(r\right)=\frac{3\pi \gamma }{k_{L}^{2}\;\left(\omega_{L}-\omega_{0}+i\Gamma \right)}n(r).$$ (9)

In principle, the solution obtained from GDT provides a good approximation only in the region where the optical potential is nonvanishing [19]. Nevertheless, it provides also a good approximation for calculating the scattering cross-sections using the formulae derived in Ref. [19] in the limit when *a* ≥ *λ*. Equation (8) can not be considered as a generally correct asymptotic form of the field. It is worth realizing, however, that it has a similar form to the solution of the Maxwell equation obtained with a slowly varying envelope approximation for the incident laser beam, with a polarization source given by a classical distribution of two-level atoms with a density *n*(***r***) [20]. In this sense, the optical potential can be interpreted as the polarization related to a two-level medium (with *Γ* = *γ*).

Both results as given above can be put in a more transparent form, namely the near field object plane field distribution can be written as

$$E_{o}\left(\rho \right)=\varepsilon_{0}\left(\rho \right)J\left(\rho \right),$$ (10)

where *ε*_0_ (***ρ***) is the probe field amplitude, and assumed to be a Gaussian beam ∝ exp(−*ρ*^2^/*ρ*_0_^2^) with width *ρ*_0_ = 1.5 mm, and J(ρ) is the transmission function of the cloud. By doing so, we have expressed the scattering through the condensate as the transmission over an optical element (BEC) whose transmittance is given by J(ρ) (we have neglected the free propagating phase factor $e^{ik_{L}z}$ here). This approximation requires the slowly varying scaled near field distribution to attain a stable asymptotic form at distance *z_a_* of the order of the size of the condensate, i.e., much smaller than the distance in which the image plane is located. Mathematically, this is equivalent to

$$\begin{array}{c} J\left(\rho \right)∼\psi \left(\rho ,z_{a}\right)\approx \psi \left(\rho ,\infty \right),\\ a\leq z_{a}\ll \text{observation}\;\text{distance}\;\text{scale}\;\left(d_{1},d_{2},f\right). \end{array}$$ (11)

In the on-shell approximation, we obtain

$$\begin{array}{c} J_{\mathrm{OS}}\;\left(\rho \right)\approx 1+\sum_{l=0}^{\infty }\frac{2l+1}{2}B_{l}\left(k_{L}\right)\\ \times \int_{0}^{\pi }\sin\theta \;d\theta J_{0}\;\left(k_{L}\rho \;\sin\theta \right)\;P_{l}\;\left(\cos\theta \right), \end{array}$$ (12)

where we have approximated cos*θ* − 1 ≈ 0 in the near field. In order to make specific calculations we need to know the atomic density profile *n*(***r***). In the following we shall use a spherically symmetric Gaussian profile given by

$$n\left(r\right)=\frac{H}{{\left(2\pi a^{2}\right)}^{3/2}}\;\exp\;\left(-\frac{r^{2}}{2a^{2}}\right).$$ (13)

Such a profile is in fact quite appropriate for the case of atoms with a negative scattering length [9, 12]. For the case of a positive scattering length the density profile can be determined from the solutions of the (mean-field) Gross-Pitaevski equation (Ref. [21], see also Refs. [9, 13]). As we have demonstrated in Ref. [13], however, using a Gaussian profile with an appropriately adjusted width leads to quantitatively similar results for scattered light.

Using the density profile given by Eq. (13) we obtain

$$\gamma_{L}^{l}=N\gamma 3\sqrt{2\pi }e^{-k_{L}^{2}a^{2}}I_{l+1/2}\left(k_{L}^{2}a^{2}\right)/\left(2k_{L}a\right),$$ (14)

where *I_l_* denotes a modified Bessel function. Within the framework of the GDT the transmission function is given by

$$\begin{array}{c} T_{\mathrm{GDT}}\;\left(\rho \right)\\ =\exp\left[-i\frac{\gamma_{\mathrm{eff}}}{\omega_{L}-\omega_{0}+i\Gamma }\;\exp\;\left(-\frac{\rho^{2}}{2a^{2}}\right)\right]. \end{array}$$ (15)

We can analogously obtain an expression for the scaled near field

$$\psi_{\mathrm{GDT}}\left(\rho ,z\right)=\exp\left[-\frac{i\gamma_{\mathrm{eff}}}{\omega_{L}-\omega_{0}+i\Gamma }\;\exp\;\left(-\frac{\rho^{2}}{2a^{2}}\right)\;\frac{1}{2}\left[1+Erf\left(z\right)\right]\right],$$ (16)

where *Erf* denotes the error function. We stress once again that more general density profiles, including the asymmetric ones as well as those obtained from mean field Gross-Pitaevskii equation for BEC can be easily implemented into the present framework [13]. We will not discuss these issues here, since we expect such profiles lead to qualitative and quantitative features of the scattered radiation very similar to those obtained with the Gaussian profile Eq. [(13)].

**Fig. 1:**
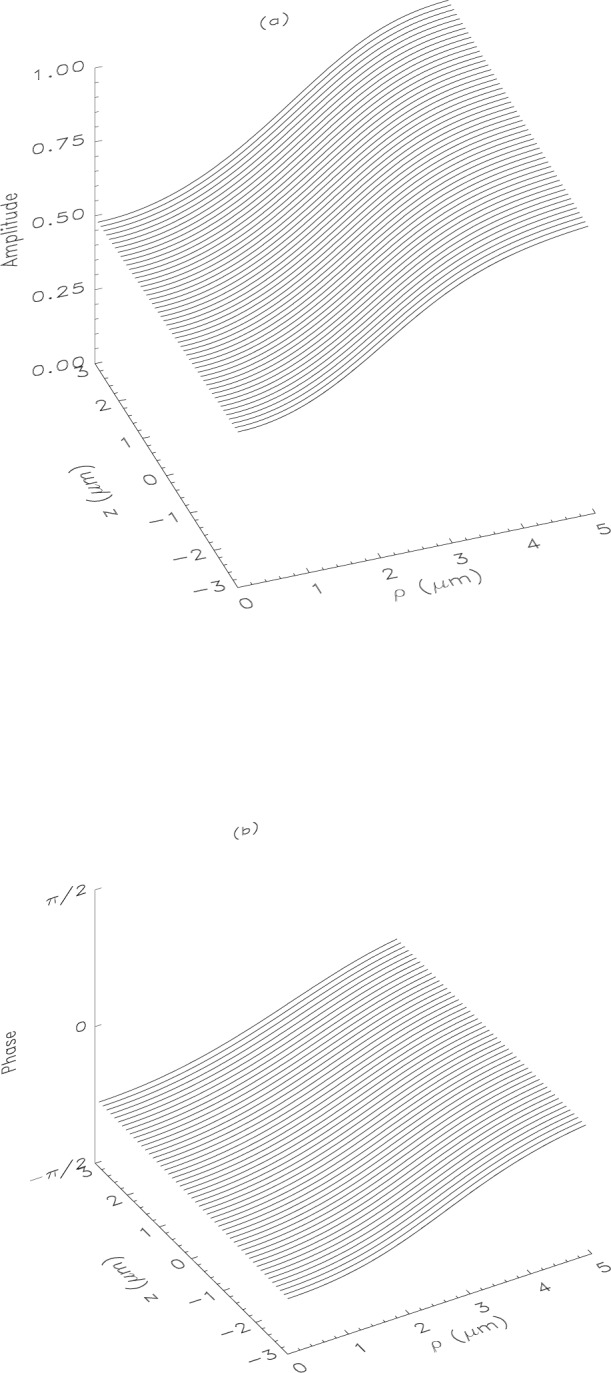
Near field distribution obtained from the on-shell approximation given by Eq. (5) for *δ* = 6*γ* = (2*π*) 1.5 × 10^7^ MHz. The rest of the parameters is given in the text. The (maximum) on-axis (*ρ* = 0) optical density is about 0.5; (a) the amplitude, (b) the phase of *ψ*_OS_(*ρ*, *z*) respectively.

**Fig. 2:**
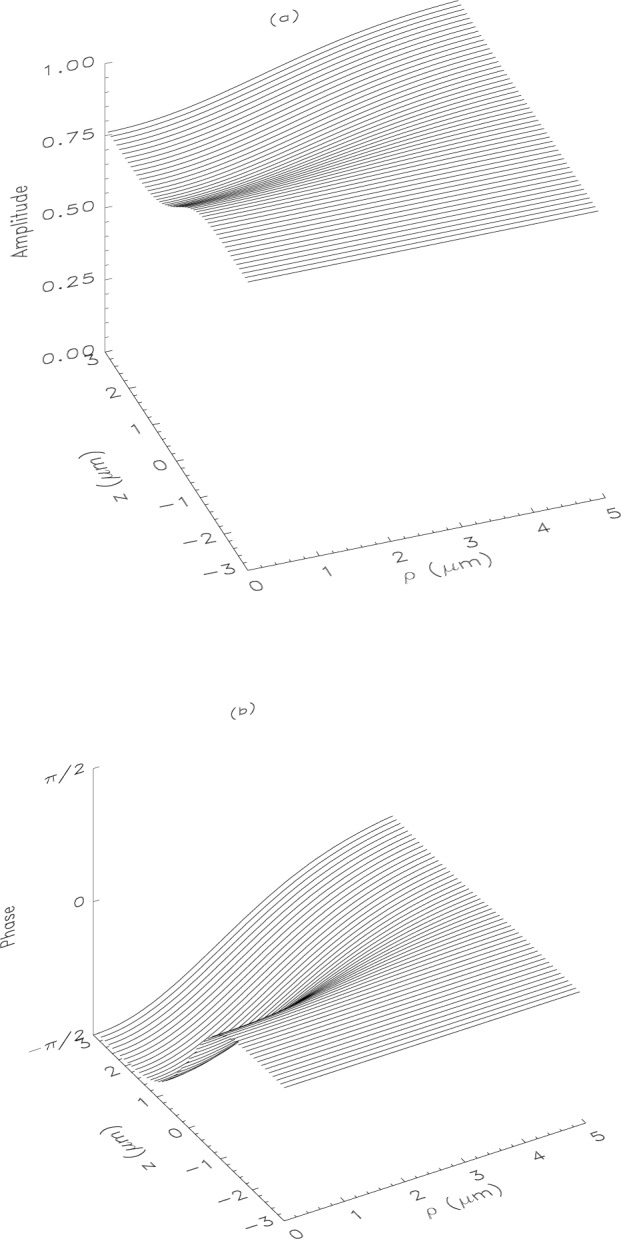
Same as in Fig. 1, except that obtained from the GDT given by Eq. (15).

From Eq. (13) it follows that the BEC is centered at ***r*** = 0. The near field patterns around the center as given by Eqs. (6) and (16) are presented in Figs. 1 and 2.

**Fig. 3:**
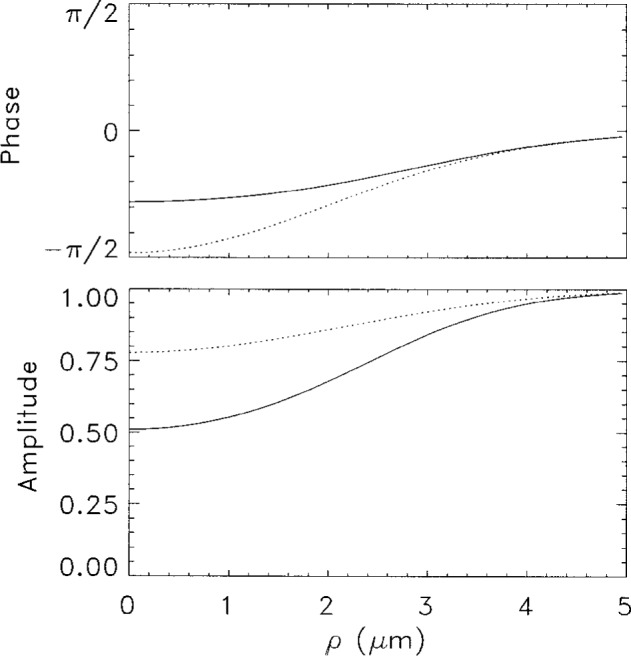
The transmission functions obtained from the *on-shell* approximation Eq. (11) (solid line) and from the GDT Eq. (13) (dotted line). Both the phase and the amplitude are displayed for *δ* = 6*γ*.

As we can see, already in a distance of the order of a few *a* beyond the condensate, stable asymptotic field patterns get established. Thus we can approximate the object plane to be also at ***r*** = 0. It should be pointed out that the near field patterns in Figs. 1 and 2 differ quite significantly. The GDT result exhibits a decrease of the amplitude and a phase shift close to the optical axis (for *ρ* ≃ 0) as *z* grows. The result of the on-shell approximation, on the other hand, varies in this regime much more slowly. In Fig. 3 we compare the results for the transmission function as given by Eqs. (12) and (15). Here, the off-axial variation of the GDT result (dashed line) is less significant than the variation of the result of the on-shell approximation (solid line). We attribute these differences to the fact that the GDT describes in fact more accurately the gradual absorption and related refraction along the optical axis. The on-shell approximation reconstructs well the field inside or after the medium only, when the absorption has already taken place. As we shall see below, the differences between the two approaches decrease essentially in the process of optical imaging.

In calculations we have used the following values of the parameters: *λ* = 0.7 μm (0.78 μm for ^87^Rb and 0.67 μm for ^7^Li), *N* = 2000, *Γ* = *γ* = (2*π*) 2.5 Mhz, and the trap size *a* = 2 μm. The results enable us to make qualitative and to some extent quantitative comparisons with the experimental results obtained for ^87^Rb and ^7^Li atoms respectively [1, 3].

## 3. Imaging Formation Process

A (coherent) imaging system converts the near field pattern *E*_o_(*ρ*) in the object plane into a new distribution *E*_i_ (*ρ*) in the image plane. In this study the optical setup involves a set of compound (ideal) lens free of abberations or any other kind of anomalies. The lens has a focal length *f*, and is located in a distance *d*_1_ away from the object plane (i.e., from the atomic cloud), and in a distance *d*_2_ away from the image plane (CCD camera). If the object is in the focus, then according to elementary optics *d*_2_ is determined by 1/*d*_1_ + 2/*d*_2_ = 1/*f*. The lens are accompanied by an aperture of diameter *D* in front.

It is reasonable to assume that the whole imaging process is paraxial, and can be treated using the Fresnel diffraction theory [22]. The magnification of the above described system is *M*_⊥_ = *d*_2_/*d*_1_. Since a magnifying optical system is not translationally invariant, the calculation of *E*_i_(*ρ*) from *E*_o_(*ρ*) has to proceed in two steps. First, we consider a magnification of the object neglecting the inversion of the image. Then we blur the image with an on-axis impulse response function (or, in another words, the Green’s function) of the imaging system [22].

The derivation of the impulse response function is straight forward by following the propagation of the phase front of the field. An on-axis impulse in the object plane is propagated in free space in the Fresnel limit over a distance *d*_1_ to the face of the lens. It is then multiplied by a spatial filtering profile of the pupil function due to the aperture, as well as an ideal phase retardation due the lens. This is followed again by a free space paraxial propagation up to the image plane. The impulse response function in such a situation is [22]

$$G_{F}\left(\rho -\rho \prime \right)=G_{0}\;\frac{2J_{l}\left(\pi D\left|\rho -\rho \prime \right|/\lambda d_{2}\right)}{\pi D\left|\rho -\rho \prime \right|/\lambda d_{2}},$$ (17)

where

*G*_0_ = *πD*^2^/(4*λ*^2^ *d*_1_*d*_2_), and *J*_1_ is the Bessel function of order 1. The spatial resolution of this impulse response function in the image plane is *ρ*_s_ ≈ 1. 22*λ d*_2_/*D*.

Finally, the image can be constructed from

$$\begin{array}{c} E_{i}\left(\rho \right)=\frac{1}{M_{⊥}^{2}}\int d\rho \prime E_{o}\left(\rho \prime /M_{⊥}\right)\;G_{F}\left(\rho -\rho \prime \right)\\ =\frac{1}{M_{⊥}^{2}}\int d\rho \prime \varepsilon_{0}\left(\rho \prime /M_{⊥}\right)J\left(\rho \prime /M_{⊥}\right)G_{F}\left(\rho -\rho \prime \right). \end{array}$$ (18)

where the prefactor $1/M_{⊥}^{2}$ is from the reduction of image intensity due to magnification.

The values of the parameters involved in the imaging process were chosen by us in such a way so that they correspond to the values reported in the Rice experiment [3] with *f* = 12 cm, *D* = 3 cm, *d*_1_ = 17 cm, and *d*_2_ = 40. 8 cm. The magnification of the system is therefore *M*_⊥_ = 2.4, and the resolution of the impulse response function is *ρ*_s_ = 11.61 μm in the image plane, which corresponds to a resolving power of ${\bar{\rho }}_{s}=\rho_{s}/M_{⊥}=3.7$ μm in the object plane. From these numbers, we conclude that any structures of order less than ${\bar{\rho }}_{s}$ in the object plane would correspond to a point source as far as the imaging properties are concerned.

## 4. Results and Discussions

We have used Eq. (18) to study the diffraction pattern formation. The numerical calculation can be done in two ways, either as a direct two-dimensional integration of Eq. (18) in terms of *x*′ and *y*′ [*ρ*′ = (*x*′, *y*′)], or by utilizing the Fourier transform technique. In the latter case, fast Fourier transform (FFT) algorithms can be used to compute the transforms for *E*_o_(*ρ*) and *G*_F_(*ρ*) separately before the inverse of their product is made to yield the result. Both FFT and explicit integration methods were tested by us.

Using Babinet’s principle [22], we can also calculate the shadow image $E_{i}^{c}$ as generated by the complementary transmission function $J^{c}=1-J$. This provides an alternative numerical scheme which is more suitable, since now the integration in Eq. (18) is basically limited to the region of the cloud.

**Fig. 4:**
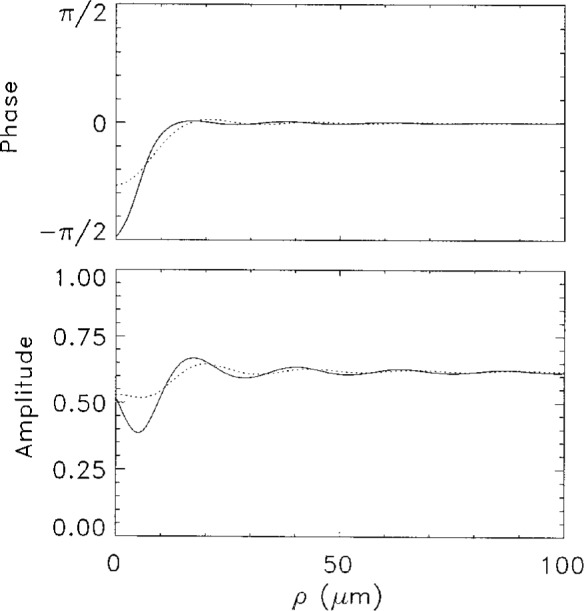
The image plane field distribution obtained from the *on-shell* approximation (solid line) and from the GDT (dotted line). Both the phase and the amplitude are displayed for *δ* = 6*γ*.

In Fig. 4 we present the field distribution in the image plane. As we mentioned, imaging reduces the differences between the on-shell approximation and the GDT results. Both approaches give now qualitatively similar results. In particular, the phase of *E*_i_(***ρ***) grows from negative values to zero for *ρ* of the order of *ρ*_s_, and remains practically constant then. The amplitude of the field exhibits characteristic oscillations.

In actual CCD image measurements one measures the relative absorption coefficient, which can be defined as

$$A\left(\rho \right)=1-\frac{{\left|E_{i}\left(\rho \right)\right|}^{2}}{{\left|E_{i}\left(\rho \right)+E_{i}^{c}\left(\rho \right)\right|}^{2}},$$ (19)

where $E_{i}\left(\rho \right)+E_{i}^{c}\left(\rho \right)$ represent the image of the probe without the presence of the cloud.

**Fig. 5:**
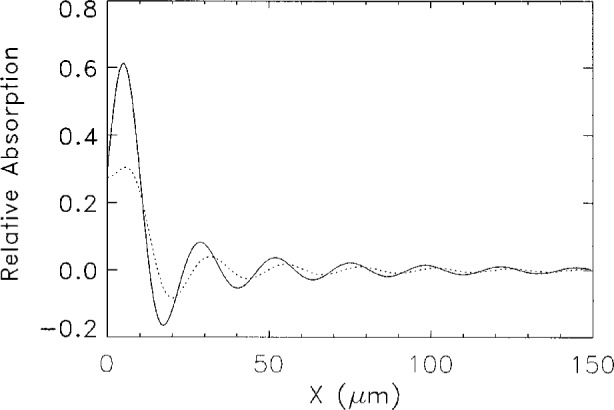
The relative absorption as given by Eq. (18) calculated from the *on-shell* approximation (solid line) and the GDT (dotted line). For *δ* = 6*γ*.

The relative absorption coefficients for the on-shell (solid line) and GDT (dashed line) approximations is shown in Fig. 5. This figure can be directly compared to Fig. 2 of Ref. [3]. The qualitative agreement in both cases is satisfactory. Quantitatively, the GDT result is closer to experimental data, giving very similar values of the relative absorption coefficient. We attribute this result to the fact that GDT seems to describe absorbtion and refraction within the medium in a more accurate manner. The oscillation period in Fig. 5 for both results of the GDT and the on-shell approximation is, however, slightly shorter than that observed by Bradley et al. [3].

## 5. Conclusions

We have studied formation of diffraction patterns in the near-resonant imaging of trapped cold atomic samples. We showed that spatial imaging similar to the spectral measurement of the coherent Rayleigh scattering [13] can probe the detailed information of the trapped atomic clouds, and may provide direct evidence of the presence of the Bose-Einstein condensate with improved imaging optics. We have used the on-shell approximation and the Glauber’s generalized diffraction theory to study light scattering off BEC and to construct near field distributions. Optical imaging theory was used to construct the field distributions in the image plane. The results compare very well with the recent experiments, and should be useful for the experimenters involved in BEC research.
